# Awareness, attitudes, barriers, and knowledge about evidence-based medicine among family physicians in Croatia: a cross-sectional study

**DOI:** 10.1186/s12875-020-01162-5

**Published:** 2020-05-16

**Authors:** Danijel Nejašmić, Davorka Vrdoljak, Valerija Bralić Lang, Josip Anđelo Borovac, Ana Marušić

**Affiliations:** 1 Department of Medical Physics and Biophysics, University of Split School of Medicine, Šoltanska 2, 21000 Split, Croatia; 2 Cochrane Croatia, University of Split School of Medicine, Šoltanska 2, Split, Croatia; 3 Department of Family Medicine, University of Split School of Medicine, Šoltanska 2, Split, Croatia; 4 Department of Family Medicine, Zagreb University School of Medicine, School of Public Health ‘Andrija Štampar’, Rockefellerova 4, Zagreb, Croatia; 5 Department of Pathophysiology, University of Split School of Medicine, Šoltanska 2, Split, Croatia; 6 Department of Research in Biomedicine and Health, University of Split School of Medicine, Šoltanska 2, Split, Croatia

**Keywords:** Evidence-based medicine, Family physicians, Awareness, Education, Quality of health care

## Abstract

**Background:**

Evidence-based medicine (EBM) aims to assist physicians in making medical decisions based on the integration of the current best evidence, clinical expertise, and patients’ values. Extensive research has been conducted regarding physicians’ awareness, attitudes, barriers, and knowledge about EBM. In Croatia, there is a lack of research on this topic, especially among family physicians (FP). The aim of this study was to assess the awareness, attitudes, barriers, and knowledge about EBM among FPs in Croatia after six years of educational activities organized and provided by Cochrane Croatia.

**Methods:**

In a cross-sectional study, conducted in 2016, we offered to FPs in Croatia a printed or online validated questionnaire to assess attitudes toward and barriers when considering the use of EBM, awareness about sources of evidence, and their level of understanding of evidence-based medicine terminology. The physicians were approached during mandatory continuing medical education courses and through their professional associations. We compared results from two groups of physicians, one with family medicine specialization and the other without.

**Results:**

295 (14%) of all officially registered FPs responded to the questionnaire. Respondents were very positive toward the promotion and usage of EBM. 160 (67%) indicated that they did not have access to the Cochrane Library. The majority reported lack of time available for finding evidence (80%), and patients’ unrealistic expectations that influence doctors’ choice of treatment (72%). Between the two groups of physicians, more family medicine specialists reported time restrictions for finding evidence. The highest level of EBM terminology understanding was reported for study design terms, and the lowest for statistical terms.

**Conclusions:**

This study demonstrated that FPs in Croatia had very positive attitudes toward the use of EBM, they agreed that EBM improves patient care, and they estimated that more than two thirds of their practice is EBM-based. Compared to the results of the first assessment of physicians in 2010, there was some increase in the level of EBM awareness among FPs. However, to further increase the quality of EBM practice in Croatia better access to EBM sources and further educational activities are needed.

## Background

Evidence-based medicine (EBM) is a paradigm for medical practice [1] where medical decisions are based on the integration of the current best evidence, clinical expertise and patients’ values [2, 3]. Over the last three decades, the concept of EBM was introduced and developed; it is now widely accepted among health professionals. The most recognized organization in the world which provides high quality EBM information is the Cochrane [1–3].

Regarding physicians and EBM, generally many studies indicate physicians’ positive attitudes towards the EBM and agree that practicing EBM improves patient care [4–10]. However, only about 50% of the physicians rated their clinical practices to be typically evidence-based [5, 11, 12]. It has been reported that more than half of physicians disagree with the notion that EBM is of limited value in primary care [5, 12]. A systematic review from 2013 also described physicians’ positive attitudes regarding EBM, and reported various EBM facilitators such as: respectful and reciprocal communication among doctors, positive attitudes of staff towards EBM, having supervisors as a point of reference for residents, etc. [13]. Physicians with prior EBM training showed a significantly more positive attitude towards EBM [14].

Regarding the knowledge of EBM, a systematic review from 2013 indicated differences in EBM application among physicians which depended on what they had learned during medical education, their confidence in current management of patients’ conditions, their perceived fit of the evidence with local facilities, etc. [13]. A study from Iran concluded that the knowledge score about EBM was higher in physicians with previous research experience and prior EBM training [14].

Although physicians in general have positive attitude towards the EBM [4–10, 13], many studies reported barriers to practicing EBM, especially among family physicians [15–18]. A recent systematic review [16] identified barriers to EBM practice related to evidence (lack of or too much available evidence; inadequate evidence), preferences and expertise of doctors (doubting the usefulness of EBM; personal experience; lack of knowledge), patients’ preferences (quality of the relationship with a patient; patients’ expectations and wishes) and family physicians (FP) setting (applicability of EBM regarding primary care patients and research population; busy workload; lack of managerial or institutional support; cost-effectiveness of EBM in practice).

A study about EBM awareness and knowledge among physicians (family physicians and hospital doctors) in Croatia published in 2010 reported low awareness about EBM and the Cochrane Library among all physicians (30%), and called for educational interventions [4]. Although medical students in Croatia have formal EBM education included in undergraduate or postgraduate programs [19], Cochrane Croatia organizes educational activities in the promotion of EBM [20]. These activities include promotion of EBM and Cochrane systematic reviews on family medicine meeting, online workshops on Cochrane systematic reviews (accredited by the Croatian Medical Chamber) and an annual Croatian Cochrane Symposium for health care workers.

The aim of this study was to assess, for the first time, the awareness, attitudes, barriers, and knowledge about EBM using a standardized questionnaire among family physicians in Croatia after six years of educational activities organized and provided by Cochrane Croatia.

## Methods

### Study design and settings

A cross-sectional survey was performed among FPs employed in primary care in Croatia. The primary health care system in Croatia is organized as a list-based system in which patients register with a single FP. There are two types of family physicians in Croatian health care – the first group consists of physicians who have completed only medical school (in this manuscript referred to as family doctors, FD), and the second group of physicians who specialized in family medicine after medical school by completing an accredited four-year family medicine residency (in this manuscript referred to as family medicine specialists, FMS). According to the official data from the Central Health Information System of Croatia, there were 2154 family physicians in 2016, with 1110 (52%) specialists [21]. The participants were recruited in two ways: during mandatory continuing medical education courses (CME) where they were invited to participate and were handed the printed version of the questionnaire, or via e-mail notification which contained a link to the web version of the questionnaire. Online version of the questionnaire was set up via the SurveyMonkey® platform (SurveyMonkey Inc., Palo Alto, CA). The software did not collect respondents’ IP addresses and was completely anonymous. Professional societies of FPs (“KoHOM - Coordination of Croatian Family Medicine” and “HUOM – Croatian Association of Family Medicine”) distributed the online version of the questionnaire among their members. Participation in the survey was voluntary, and all participants were instructed not to fill the survey more than one time. The survey was conducted between April and September 2016. Due the different types of recruitment, we were not able to calculate the actual response rate but made the estimation according to the official number of employed FPs in Croatia.

### Questionnaire

The questionnaire was based on the questionnaire developed for family physicians [11], with an additional set of questions regarding sociodemographic, professional, and practice data adapted to the Croatian health care system. In order to investigate the understanding of technical terms used in EBM, we also used the questionnaire created by McColl et al. [11]. EBM terms were organized in three groups, related to study design, statistics, and epidemiology. Each participant self-assessed his or her understanding of EBM terms. The questionnaire was piloted among 10 medical doctors and researchers affiliated with Cochrane Croatia, who tested the questionnaire to verify content, criterion-related and construct validity of the questionnaire. The questionnaire was in the Croatian language (English translation in the Supplementary material).

### Data analysis

All data analyses were performed using IBM® SPSS Statistics for Windows® (version 23.0, IBM, Armonk, NY). The distribution of quantitative data was tested by Kolmogorov-Smirnov test. Quantitative data were presented as median and interquartile range (IQR). Qualitative data were presented as absolute and relative frequencies. Depending on the data, we performed Mann-Whitney U test or χ^2^-test to compare the responses between FMSs and FDs. The level of significance (P) was 2-tailed and *P* values < 0.05 were considered statistically significant.

## Results

### Participants

In total, we had 295 (14%) respondents, with a predominance of women, 187 (79%). There were 146 (61%) FMSs and 91 (39%) FDs. The median age was 45 (33–53), with the significant difference between FMSs (50 (42–55) years) and FDs (32 (28–41) years). FMSs also had more work experience (24 (15-29) years) compared to FDs (6 (2-15) years). The participants mostly used computers at work for assessing health records of patients, issuing electronic medical prescription and referrals and for acquiring laboratory results of their patients (Table 1).

**Table 1:** Participants’ characteristics

Variable	Family medicine specialist	Family doctor	Total
**Age (years)**^b^	50 (42–55)	32 (28–41)	45 (33–53)
**Sex**^c^			
Men	31 (63.3)	18 (36.7)	49 (20.8)
Women	114 (61)	73 (39)	187 (79.2)
**Work experience (years)**^b^	24 (15–29)	6 (2–15)	19 (6.75–27)
**Computer usage at medical office**^**a,**c^			
Managing patients’ bills	82 (63.6)	47 (36.4)	129 (54)
Accounting services for my office	48 (61.5)	30 (38.5)	78 (32.6)
Health records of my patients	146 (61.6)	91 (38.4)	237 (99.2)
Electronic medical prescriptions	144 (61.8)	89 (38.2)	233 (97.5)
Electronic medical referrals	144 (62.3)	87 (37.7)	231 (96.7)
Scheduling patient’ appointments	110 (68.7)	50 (31.2)	160 (66.9)
Acquiring laboratory results of my patients	145 (62.5)	87 (37.5)	232 (91.1)
Acquiring specialized work-up results of my patients	115 (62.2)	70 (37.8)	185 (77.4)
Other^c,d^	56 (61.9)	25 (30.9)	81 (33.9)

^a^Open ended question

^b^Median (IQR)

^c^No. (%)

^d^Most common answers: e-mail communication; Internet; searching for the medical information, guidelines, and literature; visiting web pages of Agency for Medicinal Products and Medical Devices of Croatia; searching for the drug information; online education; writing various reports

### Awareness and attitudes of FPs about EBM

The participants had very positive attitude towards the promotion of EBM, and thought that EBM improves patient care delivery. They were also very positive about the attitudes of their colleagues toward the EBM, and reported that research findings were extremely useful in their daily management of patients. They estimated that about 70% of their clinical practice is based on EBM. They disagreed with the statement that EBM is of limited value in general practice due to lack of scientific foundation in primary care. However, the participants were neutral in regard to the statement that adoption of EBM imposes additional burden on already overloaded FPs (Table 2). There were no significant differences between FMSs and FDs for this part of the questionnaire.

**Table 2:** Responses regarding evidence-based medicine

Question/Statement	Family medicine specialist^**†**^	Family doctor^**a**^	***P***-value^**b**^
How would you describe your attitude towards the current promotion of evidence-based medicine?	5 (4–5)	5 (4–5)	0.396
How would you describe the attitude of most of your GP colleagues towards evidence-based medicine?	4 (3–4)	4 (3–4)	0.992
How useful are research findings in your day-to-day management of patients?	4 (4–5)	4 (4–5)	0.289
What percentage of your clinical practice do you feel is currently evidence-based?	70% (70–80%)	70% (60–80%)	0.203
Practicing evidence-based medicine improves patient care.	5 (4–5)	5 (4–5)	0.659
Evidence-based medicine is of limited value in general practice because much of primary care lacks a scientific base.	1 (1–3)	2 (1–3)	0.174
The adoption of EBM, however worthwhile as an ideal, places another demand on already overloaded GPs.	3 (2–4)	3 (2–4)	0.097

^a^Median (IQR)

^b^Mann-Whitney U test

The majority of participants (160 (67%)) reported that they did not have access to The Cochrane Library. Among those who had access, there were significantly more FMSs that were accessing The Cochrane library at home or at their medical office (χ^2^-test, *p* = 0.007). Although almost all participants (225 (95%)) declared that they were using online sources of medical information, the frequency of using the evidence to solve a problem in clinical practice for 203 (89%) participants was three times or less in the last three months. Our participants preferred using the evidence prepared by medical associations of interest, online summaries, and guidelines than assessing original articles or systematic reviews and meta-analysis. In general, the participants had positive attitude toward the different sources of evidence (Table 3).

**Table 3:** Responses regarding sources of the evidence (No., %)

Question	Family medicine specialist	Family doctor	Total	***P***-value^**a**^
**Do you have an access to Cochrane library?**				
Yes, at home	20 (66.7)	10 (33.3)	30 (12.6)	0.007
Yes, at my medical office	11 (73.3)	4 (26.7)	15 (6.3)	
Yes, both at home and at my medical office	28 (84.8)	5 (15.2)	33 (13.9)	
No	87 (54.4)	73 (45.6)	160 (67.2)	
**Do you use online sources of medical information available through online journals and guidelines made by medical associations of interest?**				
Yes	141 (62.7)	84 (37.3)	225 (94.9)	0.145
No	5 (41.7)	7 (58.3)	12 (5.1)	
**In last three months, how many times have you used an original research from a medical journal to solve a problem in your clinical practice?**				
Not even once	23 (47.9)	25 (52.1)	48 (21.1)	0.121
Once	57 (64.8)	31 (35.2)	88 (38.8)	
2–3 times	46 (68.7)	21 (31.3)	67 (29.%)	
4 times or more	16 (66.7)	8 (33.3)	24 (10.6)	
**Original articles published in high-impact journals**				
Not useful	7 (87.5)	1 (12.%)	8 (3.6)	0.131
Useful	70 (57.4)	52 (42.6)	122 (54.2)	
Very useful	63 (66.3)	32 (37.6)	95 (42.2)	
**Online sources that provide summaries of important research that is relevant for your field (EBM, Bandolier, POEMS)**				
Not useful	6 (66.7)	3 (33.3)	9 (4.1)	0.414
Useful	60 (57.1)	45 (42.9)	105 (47.3)	
Very useful	71 (65.7)	37 (34.3)	108 (48.6)	
**Systematic reviews or meta-analysis (for example: Cochrane Library)**				
Not useful	8 (72.2)	3 (27.3)	11 (4.9)	0.461
Useful	63 (58.9)	44 (41.4)	107 (48)	
Very useful	69 (65.7)	36 (34.3)	105 (47.1)	
**Clinical guidelines that are founded on EBM**				
Not useful	3 (75)	1 (25)	4 (1.8)	0.742
Useful	41 (63.4)	28 (40.6)	69 (30.5)	
Very useful	97 (63.4)	56 (36.%)	153 (67.7)	
**Access to MEDLINE in your office**				
Not useful	6 (54.5)	5 (45.5)	11 (5)	0.483
Useful	60 (58.8)	42 (41.2)	102 (45.9)	
Very useful	72 (66.1)	37 (33.9)	109 (49.1)	
**A librarian that performs literature search on certain topic of interest, per my request**				
Not useful	38 (64.4)	21 (35.6)	59 (27.1)	0.704
Useful	70 (61.9)	43 (38.1)	113 (51.8)	
Very useful	26 (56.5)	20 (43.5)	46 (21.1)	
**Editorial of a Journal that sends me an article per my request**				
Not useful	25 (56.8)	19 (43.2)	44 (20)	0.252
Useful	88 (66.7)	44 (33.3)	132 (60)	
Very useful	24 (54.5)	20 (45.5)	44 (20)	
**Seminars and workshops for family medicine doctors about literature search and critical appraisal of evidence**				
Not useful	5 (55.6)	4 (44.4)	9 (4)	0.903
Useful	78 (62.9)	46 (37.1)	124 (55.1)	
Very useful	58 (63)	34 (37)	92 (40.9)	

^a^χ^2^-test

### Barriers in the use of EBM by FPs

Regarding the barriers in using EBM, the majority of participants reported the lack of time for finding evidence (188 (80%)), reading and assessing evidence (186 (79%)), discussing research results with the patients (190 (81%)), as well as patients’ unrealistic expectations that influence doctors’ choice of treatment (168 (72%)). FMSs more often reported lack of time for finding evidence as a barrier towards using EBM compared to FDs (χ^2^-test, *p* = 0.036). FMSs less often reported patients’ unrealistic expectations, and influence on choice of treatment (χ^2^-test, *p* = 0.05) or financial aspect of access to EBM sources (χ^2^-test, *p* < 0.001) as barriers to EBM practice (Table 4).

**Table 4:** Responses on barriers in using EBM (No., %)

Statement	Family medicine specialist	Family doctor	Total	***P***-value^a^
**There is not enough evidence relevant for family medicine practice**				
Not a barrier	78 (54.2)	49 (38.6)	127 (54)	0.844
Significant barrier	61 (60.4)	40 (39.6)	101 (43)	
Very significant barrier	5 (71.4)	2 (28.6)	7 (3)	
**Patients request treatments that have no proven medical efficacy**				
Not a barrier	53 (68.8)	24 (31.2)	77 (32.8)	0.234
Significant barrier	64 (56.6)	49 (43.4)	113 (48.1)	
Very significant barrier	27 (60)	18 (40)	45 (19.1)	
**I do not have enough skills for finding evidence**				
Not a barrier	81 (64.3)	45 (35.7)	126 (53.4)	0.185
Significant barrier	57 (61.3)	36 (38.7)	93 (39.4)	
Very significant barrier	7 (41.2)	10 (58.8)	17 (7.2)	
**I do not have enough time for finding evidence**				
Not a barrier	29 (63)	17 (37)	46 (19.7)	0.036
Significant barrier	88 (67.2)	43 (32.8)	131 (56)	
Very significant barrier	27 (47.4)	30 (52.6)	57 (24.4)	
**I do not have enough skills for critical assessment of evidence**				
Not a barrier	58 (63)	34 (37)	92 (39.5)	0.112
Significant barrier	79 (64.2)	44 (35.8)	123 (52.8)	
Very significant barrier	7 (38.9)	11 (61.1)	18 (7.7)	
**I do not have enough time for reading and assessment of evidence**				
Not a barrier	35 (70)	15 (30)	50 (21.2)	0.325
Significant barrier	82 (60.3)	54 (39.7)	136 (57.6)	
Very significant barrier	28 (56)	22 (44)	50 (21.2)	
**I do not have enough skills in presenting results of relevant research to my patients**				
Not a barrier	69 (63.9)	39 (36.1)	108 (46)	0.722
Significant barrier	62 (59.6)	42 (40.4)	104 (44.3)	
Very significant barrier	13 (56.5)	10 (43.5)	23 (9.8)	
**I do not have enough time to discuss research results with my patients during their scheduled appointment with me**				
Not a barrier	32 (71.7)	13 (28.9)	45 (19.1)	0.308
Significant barrier	76 (60.8)	49 (39.2)	125 (53.2)	
Very significant barrier	37 (56.9)	28 (43.1)	65 (27.7)	
**The use of EBM will further limit the number of patients that I can examine at my medical office**				
Not a barrier	66 (66.7)	33 (33.3)	99 (42.1)	0.150
Significant barrier	66 (60)	44 (40)	110 (46.8)	
Very significant barrier	12 (46.2)	14 (53.8)	26 (11.1)	
**Despite the results of relevant studies, patients have unrealistic expectations that influence my choice of treatment**				
Not a barrier	47 (71.2)	19 (28.8)	66 (28.2)	0.05
Significant barrier	71 (60.7)	46 (39.3)	117 (50)	
Very significant barrier	25 (49)	26 (51)	51 (21.8)	
**I am concerned about the financial aspects of my practice because the access to EBM sources is costly**				
Not a barrier	64 (79)	17 (21)	81 (34.4)	< 0.001
Significant barrier	52 (56.5)	40 (43.5)	92 (39)	
Very significant barrier	29 (46)	34 (54)	63 (26.7)	

^a^χ^2^-test

### Understanding study design terms

Considering the understanding of study design terms in EBM, more than two-thirds of the participants had high level of understanding or understanding with the possibility to explain to the others. The lowest level of understanding was for a case control study (155 (66%)), and the highest was for a case report (229 (96%)). More FMSs reported higher levels of understanding for a cohort study (χ^2^-test, *p* = 0.007, Table 5).

**Table 5:** Responses on understanding terms in EBM (study design terms)

Term	Family medicine specialist	Family doctor	Total	***P***-value^a^
**Meta-analysis**				
It would not be helpful to me to understand	3 (100)	0 (0)	3 (1.3)	0.084
Don’t understand but would like to	14 (43.8)	18 (56.3)	32 (13.6)	
Basic understanding	79 (61.7)	49 (38.8)	128 (54.2)	
Yes, understand and could explain to others	48 (65.8)	25 (34.2)	73 (30.9)	
**Randomized controlled clinical trial**				
It would not be helpful to me to understand	1 (100)	0 (0)	1 (0.4)	0.077
Don’t understand but would like to	8 (36.4)	14 (63.6)	22 (9.3)	
Basic understanding	81 (63.8)	46 (36.2)	127 (53.6)	
Yes, understand and could explain to others	55 (63.2)	32 (36.8)	87 (36.7)	
**Cohort study**				
It would not be helpful to me to understand	2 (100)	0 (0)	2 (0.8)	0.007
Don’t understand but would like to	14 (36.8)	24 (63.2)	38 (16.1)	
Basic understanding	82 (65.6)	43 (34.4)	125 (53)	
Yes, understand and could explain to others	46 (64.8)	25 (35.2)	71 (30.1)	
**Case control study**				
It would not be helpful to me to understand	4 (66.7)	2 (33.3)	6 (2.6)	0.671
Don’t understand but would like to	42 (56.8)	32 (43.2)	74 (31.5)	
Basic understanding	61 (61)	39 (39)	100 (42.6)	
Yes, understand and could explain to others	37 (67.3)	18 (32.7)	55 (23.4)	
**Cross-sectional study**				
It would not be helpful to me to understand	2 (66.7)	1 (33.3)	3 (1.3)	0.646
Don’t understand but would like to	29 (53.7)	25 (46.3)	54 (22.8)	
Basic understanding	71 (63.4)	41 (36.6)	112 (47.3)	
Yes, understand and could explain to others	43 (63.2)	25 (36.8)	68 (28.7)	
**Case report**				
It would not be helpful to me to understand	2 (100)	0 (0)	2 (0.8)	0.132
Don’t understand but would like to	3 (50)	3 (50)	6 (2.5)	
Basic understanding	46 (52.9)	41 (47.1)	87 (36.7)	
Yes, understand and could explain to others	94 (66.2)	48 (33.8)	142 (59.9)	

^a^χ^2^-test

### Understanding statistical terms

The lowest understanding of statistical terms used in EBM was reported for interquartile range (76 (32%)), type I and II error (78 (33%)), and for mode (107 (45%)), while the highest level of understanding was reported for a representative sample (216 (92%)), precision and accuracy (196 (83%)), and standard deviation (194 (82%)). There was statistical difference between the groups for terms interquartile range (χ^2^-test, *p* = 0.006), standard deviation (χ^2^-test, p = 0.006), precision and accuracy (χ^2^-test, *p* = 0.002), and representative sample (χ^2^-test, *p* = 0.026) towards the better understanding of terms among FMSs. In addition, FMSs reported lower understanding of terms type I and type II errors (χ^2^-test, *p* = 0.005) (Table 6).

**Table 6:** Responses on understanding terms in EBM (statistical terms) (No., %)

Term	Family medicine specialist	Family doctor	Total	***P***-value^**a**^
**Mode**				
It would not be helpful to me to understand	14 (77.8)	4 (22.2)	18 (7.6)	0.153
Don’t understand but would like to	71 (63.4)	41 (36.6)	112 (47.3)	
Basic understanding	43 (53.1)	38 (46.9)	81 (34.2)	
Yes, understand and could explain to others	18 (69.2)	8 (30.8)	26 (11)	
**Median**				
It would not be helpful to me to understand	12 (80)	3 (20)	15 (6.3)	0.078
Don’t understand but would like to	29 (49.2)	30 (50.8)	59 (24.8)	
Basic understanding	83 (63.8)	47 (36.2)	130 (54.6)	
Yes, understand and could explain to others	23 (67.6)	11 (32.4)	34 (14.3)	
**Interquartile range (IQR)**				
It would not be helpful to me to understand	16 (66.7)	8 (33.3)	24 (10.1)	0.006
Don’t understand but would like to	73 (53.3)	64 (46.7)	137 (57.8)	
Basic understanding	39 (69.6)	17 (30.4)	56 (23.6)	
Yes, understand and could explain to others	18 (90)	2 (10)	20 (8.4)	
**Standard deviation (SD)**				
It would not be helpful to me to understand	9 (81.8)	2 (18.2)	11 (4.6)	0.006
Don’t understand but would like to	12 (37.5)	20 (62.5)	32 (13.5)	
Basic understanding	84 (61.3)	53 (38.7)	137 (57.8)	
Yes, understand and could explain to others	41 (71.9)	16 (28.1)	57 (24.1)	
**Precision and accuracy**				
It would not be helpful to me to understand	8 (100)	0 (0)	8 (3.4)	0.002
Don’t understand but would like to	14 (42.4)	19 (57.6)	33 (13.9)	
Basic understanding	83 (58.9)	58 (41.4)	141 (59.5)	
Yes, understand and could explain to others	41 (74.5)	14 (25.5)	55 (23.2)	
**Representative sample**				
It would not be helpful to me to understand	5 (100)	0 (0)	5 (2.1)	0.026
Don’t understand but would like to	5 (33.3)	10 (66.7)	15 (6.4)	
Basic understanding	84 (60)	56 (40)	140 (59.3)	
Yes, understand and could explain to others	51 (67.1)	25 (32.9)	76 (32.2)	
**Test power**				
It would not be helpful to me to understand	9 (75)	3 (25)	12 (5.1)	0.167
Don’t understand but would like to	26 (51)	25 (49)	51 (21.7)	
Basic understanding	84 (61.8)	52 (38.2)	136 (57.9)	
Yes, understand and could explain to others	26 (72.2)	10 (27.8)	36 (15.3)	
***P*-value**				
It would not be helpful to me to understand	8 (80)	2 (20)	10 (4.2)	0.358
Don’t understand but would like to	47 (56)	37 (44)	84 (35.6)	
Basic understanding	66 (64.1)	37 (35.9)	103 (43.6)	
Yes, understand and could explain to others	26 (66.7)	13 (33.3)	39 (16.5)	
**Confidence interval (CI)**				
It would not be helpful to me to understand	7 (84.5)	1 (12.5)	8 (3.4)	0.270
Don’t understand but would like to	54 (56.3)	42 (43.8)	96 (40.3)	
Basic understanding	64 (63.4)	37 (36.6)	101 (42.4)	
Yes, understand and could explain to others	22 (66.7)	11 (33.3)	33 (13.9)	
**Type I and type II errors**				
It would not be helpful to me to understand	13 (92.9)	1 (7.1)	14 (5.9)	0.005
Don’t understand but would like to	91 (62.8)	54 (37.2)	145 (61.2)	
Basic understanding	27 (46.6)	31 (53.4)	58 (24.5)	
Yes, understand and could explain to others	15 (75)	5 (25)	20 (8.4)	

^a^χ^2^-test

### Understanding epidemiological terms

The participants expressed high level of understanding the epidemiological EBM terms. The lowest level of understanding was reported for odds ratio (133 (56%)), and the highest for sensitivity and specificity of the test (219 (92%)). Between the groups, there was no statistically significant difference in understanding any of epidemiological terms (Table 7).

**Table 7:** Responses on understanding terms in EBM (epidemiological terms) (No., %)

Term	Family medicine specialist	Family doctor	Total	***P***-value^**a**^
**Odds ratio (OR)**				
It would not be helpful to me to understand	13 (72.2)	5 (27.8)	18 (7.6)	0.728
Don’t understand but would like to	54 (62.1)	33 (37.9)	87 (36.6)	
Basic understanding	61 (60.4)	40 (39.6)	101 (42.4)	
Yes, understand and could explain to others	18 (56.3)	14 (43.8)	32 (13.4)	
**Relative risk (RR)**				
It would not be helpful to me to understand	4 (57.1)	3 (42.9)	7 (2.9)	0.938
Don’t understand but would like to	23 (63.9)	13 (26.1)	36 (15.1)	
Basic understanding	94 (61.4)	59 (38.6)	153 (64)	
Yes, understand and could explain to others	26 (60.5)	17 (3.5)	43 (18)	
**Absolute risk (AR)**				
It would not be helpful to me to understand	3 (42.9)	4 (57.1)	7 (2.9)	0.712
Don’t understand but would like to	21 (58.3)	15 (41.7)	36 (15.1)	
Basic understanding	96 (63.2)	56 (36.8)	152 (63.9)	
Yes, understand and could explain to others	26 (60.5)	17 (39.5)	43 (18.1)	
**Number needed to treat (NNT)**				
It would not be helpful to me to understand	2 (50)	2 (50)	4 (1.7)	0.640
Don’t understand but would like to	28 (56)	22 (44)	50 (21)	
Basic understanding	86 (61.4)	54 (38.6)	140 (58.8)	
Yes, understand and could explain to others	30 (68.2)	14 (31.8)	44 (18.5)	
**Sensitivity and specificity of the test**				
It would not be helpful to me to understand	1 (50)	1 (50)	2 (0.8)	0.362
Don’t understand but would like to	8 (44.4)	10 (55.6)	18 (7.5)	
Basic understanding	95 (61.3)	60 (38.7)	155 (64.9)	
Yes, understand and could explain to others	43 (67.2)	21 (32.8)	64 (26.8)	
**Heterogeneity**				
It would not be helpful to me to understand	4 (57.1)	3 (42.9)	7 (2.9)	0.806
Don’t understand but would like to	34 (56.7)	26 (43.3)	60 (25.5)	
Basic understanding	80 (64)	45 (36)	125 (52.5)	
Yes, understand and could explain to others	28 (60.9)	18 (39.1)	46 (19.3)	
**Publication bias**				
It would not be helpful to me to understand	3 (60)	2 (40)	5 (2.1)	0.674
Don’t understand but would like to	57 (66.3)	29 (33.7)	86 (36)	
Basic understanding	61 (57.5)	45 (42.5)	106 (44.4)	
Yes, understand and could explain to others	26 (61.9)	16 (38.1)	42 (17.6)	
**Positive predictive value**				
It would not be helpful to me to understand	2 (40)	3 (60)	5 (2.1)	0.497
Don’t understand but would like to	40 (57.1)	30 (42.9)	70 (29.3)	
Basic understanding	79 (62.7)	47 (37.3)	126 (52.7)	
Yes, understand and could explain to others	26 (68.4)	12 (31.6)	38 (15.9)	
**Hierarchy of evidence**				
It would not be helpful to me to understand	1 (50)	1 (50)	2 (0.8)	0.104
Don’t understand but would like to	25 (51)	24 (49)	49 (20.5)	
Basic understanding	87 (60.8)	56 (39.2)	143 (59.8)	
Yes, understand and could explain to others	34 (75.6)	11 (24.4)	45 (18.8)	

^a^χ^2^-test

## Discussion

### Awareness and attitudes of FPs about EBM

To the best of our knowledge this is the first study about EBM awareness, and attitudes among family physicians in transitional countries within the southern east part of Europe, which have similar organizational structure of primary health care [22, 23]. Our study showed that family physicians in Croatia had a very positive attitude towards the use of EBM, which is consistent with previous studies [4–10, 13] and they strongly agreed that EBM improves patient care. Although the participants estimated that around 70% of their practice is EBM-based, this number could be overestimated, since the majority of participants (203 (89%)) responded that they used an original research article to solve a patient’s problem up to three times in the last three months. Rare usage of original research articles to solve a patient’s problem could be also be the reason why our participants strongly disagreed that EBM is of limited value due to the lack of evidence in general practice, which is not consistent with previous studies [16].

### Information about sources of the evidence

Our participants preferred EBM sources prepared by relevant medical associations, online summaries, and guidelines rather than assessing EBM sources personally (original articles or systematic reviews and meta-analysis), which is in line with previous studies [11, 13, 16, 18]. This could be caused by the lack of time or knowledge for reading and understanding the reports of primary studies and systematic reviews. Participants reported that systematic reviews or meta-analysis were a useful EBM source, but still two-thirds of the participants indicated that they did not have access to The Cochrane Library, which presents the first obstacle in practicing EBM.

Compared to the study published in Croatia in 2010 [4], there is an overall increase in access and use of The Cochrane Library among FPs in Croatia. Today, only medical schools and university hospitals in Croatia have free access to The Cochrane Library, via Croatian Academic and Research Network (CARNet). The use of EBM, especially The Cochrane Library, could be increased if all the FPs could become members of CARNet. Our study also indicates that there was a modest progress in some aspects in the level of EBM awareness compared to the study from 2010 which could be related to the educational activities organized by Cochrane Croatia in terms of EBM promotion. Due to the small number of FP participants and a different questionnaire used in the 2010, we could not directly compare all results between these two studies.

### Barriers in the use of EBM by FPs

Among barriers related to the FPs’ preferences and expertise (questions 4.1, 4.3, 4.5, 4.7 from the questionnaire), the participants did not report any of the barriers as significant or very significant. Only having skills for critical assessment of evidence was reported as a significant barrier. Although our findings are in line with previous studies included in a systematic review [16], this level of confidence could be the result of self-evaluation without any objective control.

This study showed that patient-related barriers (questions 4.2, 4.10 from the questionnaire) limit the use of EBM, especially when patients have unrealistic expectations that influence FPs choice of treatment. Previous studies also confirm that, in the situations where FPs choice of treatment does not match the wishes of patient, FPs could feel pressured from patients [11, 16, 17].

Regarding barriers related to the practice setting (questions 4.4, 4.6, 4.8, 4.9, 4.11), the most common barrier reported in this study and other studies is lack of time that FPs need to overcome in order to practice EBM [16].

### Differences between FMSs and FDs regarding the barriers

We found a significant difference between FMSs and FDs regarding barriers in using EBM. The lack of time for finding evidence was found as a significant barrier to EBM practice for FMSs. The lack of specialist education is likely to result in a defensive approach i.e. more frequent referral to secondary health care, which is less time-consuming [24]. Searching for evidence, appraising it, and discussing with the patient requires more time investment than simply referring a patient to a secondary health care, and this notion was demonstrated by FPs in this study. FMSs and FDs also differed in coping with unrealistic patient expectations as the main barrier to using EBM in practice, where those barriers were significantly more perceived by FDs. The aforementioned emphasizes the importance of doctors’ specialization for FPs, i.e. four-year training under the supervision of their mentors, usually family medicine specialists with high degree of experience [25, 26].

### Knowledge of FPs about EBM

Generally, the participants reported high level of understanding of all EBM technical terms, especially FPs with specialization. The highest level of understanding was reported for study design terms while the lowest understanding was shown for statistical terms. FMSs had better understanding than FDs of interquartile range (IQR), standard deviation (SD), precision and accuracy, and representative sample. The reason for these differences could be the result of specialization training of FMSs. However, general low understanding of some basic statistical terms such as mode, IQR, *P*-value, and confidence interval raises doubt in adequate interpretation and critical appraisal of evidence sources that FPs might use in their everyday practice. Overall, compared to previous studies [5, 8], our participants achieved higher scoring in self-reported understanding technical terms used in EBM.

To be able to practice EBM, FPs need to have better training in research methodology principles, especially during formal medical education and postgraduate studies that also include specialization curricula. Various EBM training approaches were studied in order to encourage EBM use in physician practice [27–33], and they should include multifaceted, clinically integrated interventions in order to improve knowledge, skills, attitudes, and behavior amongst practicing health professionals [26]. Recently, a group of authors proposed an “EBM competency framework for real-world general practice” which has been developed out of the empirical research while taking into account the constraints of today’s general practice, but further validation of this approach is needed [34].

### Limitations

Our study had several limitations. Our study sample was based on voluntary participation of FPs, and we had an unbalanced sample distribution between FPs with and without specialization (61% vs. 39%). Due to the lack of information, we could not confirm whether this distribution is similar in ratio to that of the national level. Voluntary participation in the study could have attracted more enthusiastic and motivated FPs, so that our results could be more positive compared to the whole population of employed FPs. However, we believe that a larger sample size would not greatly affect results in general. Another limitation is inherent to the survey study design. Our results could have been influenced by overestimation of FPs knowledge about EBM, because their knowledge was not assessed and only their opinion about EBM was questioned.

## Conclusions

Our study demonstrated that FPs in Croatia had very positive attitudes toward the use of EBM, and they strongly agreed that EBM improves patient care. They estimated that more than two thirds of their practice is EBM-based. This could be overestimated, since the majority of participants were not often using an original research article to solve a patient’s problem. FPs preferred EBM sources prepared by relevant medical associations, online summaries and guidelines rather than assessing EBM sources personally. We found improvement in some aspects in the level of EBM awareness, compared to the similar study conducted in 2010 among medical doctors in Croatia.

Comparing FMSs and FDs, the significant difference was found in reporting barriers (lack of time to practice EBM and coping with patients’ unrealistic expectations), and in understanding EBM technical terms where specialist education and experience could be in favor of FMSs. The many barriers that that were reported in this study were already described in previous studies among family physicians, which indicates a great difficulty to overcome as they are consistently present to their regular working environment.

Finally, one of the solutions that could enhance the knowledge, attitudes, and awareness about EBM among family physicians is to provide them with free access to the EBM databases such as The Cochrane Library in primary care institutions and to offer them more educational activities that would promote and encourage EBM use in everyday practice.

## Supplementary information

**Additional file 1.** Questionnaire.

## Data Availability

The datasets used during the current study are available from the corresponding author on reasonable request.

## Abbreviations

CI: Confidence interval
CME: Continuing medical education
EBM: Evidence-based medicine
FD: Family doctors (without specialization)
FP: Family physicians
FMS: Family medicine specialist
IQR: Interquartile range
SD: Standard deviation
