# Supplementary material for: Awareness, attitudes, barriers, and knowledge about evidence-based medicine among family physicians in Croatia: a cross-sectional study
Source: BMC Fam Pract. 2020 May 16;21:88. doi: 10.1186/s12875-020-01162-5 (PMC7231414; doi:10.1186/s12875-020-01162-5)
Supplement: Supplementary file 1 — Additional file 1. Questionnaire. [file 12875_2020_1162_MOESM1_ESM.docx]

**QUESTIONNAIRE**

| **1. Evidence-based medicine (*EBM*)** | | | | | | | | | | | | | | | | | |
| --- | --- | --- | --- | --- | --- | --- | --- | --- | --- | --- | --- | --- | --- | --- | --- | --- | --- |
| *Select one number on the scale from 1-5 or percentage (%) which correspondes to your opinion.* | | | | | | | | | | | | | | | | | |
| 1.1 How would you describe your attitude towards the current promotion of evidence-based medicine? | | | | | | | | | | | | | | | | | |
| *Extremely negative* | | | | | | | 1 | | 2 | 3 | | 4 | 5 | | *Extremely positive* | | |
| 1.2 How would you describe the attitude of most of your GP colleagues towards evidence-based medicine? | | | | | | | | | | | | | | | | | |
| *Extremely negative* | | | | | | | 1 | | 2 | 3 | | 4 | 5 | | *Extremely positive* | | |
| 1.3 How useful are research findings in your day to day management of patients? | | | | | | | | | | | | | | | | | |
| *Totally Useless* | | | | | | | 1 | | 2 | 3 | | 4 | 5 | | *Extremely Useful* | | |
| 1.4 What percentage of your clinical practice do you feel is currently evidence-based? | | | | | | | | | | | | | | | | | |
|  | 0% | 10% | 20% | 30% | 40% | 50% | | 60% | | | 70% | | | 80% | | 90% | 100% |
| 1.5 Practicing evidence-based medicine improves patient care. | | | | | | | | | | | | | | | | | |
| *Strongly Disagree* | | | | | | | 1 | | 2 | 3 | | 4 | 5 | | *Strongly Agree* | | |
| 1.6 Evidence-based medicine is of limited value in general practice because much of primary care lacks a scientific base. | | | | | | | | | | | | | | | | | |
| *Strongly Disagree* | | | | | | | 1 | | 2 | 3 | | 4 | 5 | | *Strongly Agree* | | |
| 1.7 The adoption of EBM, however worthwhile as an ideal, places another demand on already overloaded GPs. | | | | | | | | | | | | | | | | | |
| *Strongly Disagree* | | | | | | | 1 | | 2 | 3 | | 4 | 5 | | *Strongly Agree* | | |

| **2. Professional information** | |
| --- | --- |
| 2.1 For which activities do you use computer at your medical office??  *(Select one or more answers)* | 1. Managing patient' bills |
|  | 2. Accounting services for my office |
|  | 3. Health records of my patients |
|  | 4. Electronic medical prescriptions |
|  | 5. Electronic medical referrals |
|  | 6. Scheduling patient' appointments |
|  | 7. Acquiring laboratory results of my patients |
|  | 8. Acquiring specialized work-up results of my patients |
|  | 9. Something else? *(write here)* |
| 2.2 Do you have an access to Cochrane library? | 1. Yes, at home. |
|  | 2. Yes, at my clinic. |
|  | 3. Yes, at home and at my clinic. |
|  | 4. No. |

| **3. Information about sources of the evidence** | | | | | | | |
| --- | --- | --- | --- | --- | --- | --- | --- |
| *Select one of the possible answers.* | | | | | | | |
| 3.1 Do you use online sources of medical information available through online journals and guidelines made by medical associations of interest? | YES | | NO  *(if, „No“, go to the section*  *"4. Barriers to EBM application")* | | | | |
| 3.2 In last three months, how many times have you used an original research from a medical journal to solve a problem in your clinical practice? | Not even once | Once | 2 - 3 times | | 4 times or more | | |
|  | | | | | | | |
| Evaluate the usefulness of following sources of medical information:  *(Choose one of possible three answers and cross "x" in appropriate box for each source)* | | | | Not useful | | Useful | Very useful |
| 3.3 Original articles published in high-impact journals | | | |  | |  |  |
| 3.4 Online sources that provide summaries of important research that is relevant for your field (EBM, Bandolier, POEMS) | | | |  | |  |  |
| 3.5 Systematic reviews or meta-analysis (for example: Cochrane Library) | | | |  | |  |  |
| 3.6 Clinical guidelines that are founded on EBM | | | |  | |  |  |
| 3.7 Access to MEDLINE in your office | | | |  | |  |  |
| 3.8 A librarian that performs literature search on certain topic of interest, per my request | | | |  | |  |  |
| 3.9 Editorial of a Journal that sends me an article per my request | | | |  | |  |  |
| 3.10 Seminars and workshops for family medicine doctors about literature search and critical appraisal of evidence | | | |  | |  |  |

| **4. Barriers in using EBM**  *(Choose one of possible three answers and cross "x" in appropriate box for each barrier)* | Not a barrier | Significant barrier | Very significant barrier |
| --- | --- | --- | --- |
| 4.1 There is not enough evidence relevant for family medicine practice |  |  |  |
| 4.2 Patients request treatments that have no proven medical efficacy |  |  |  |
| 4.3 I do not have enough skills for finding evidence |  |  |  |
| 4.4 I do not have enough time for finding evidence |  |  |  |
| 4.5 I do not have enough skills for critical assessment of evidence |  |  |  |
| 4.6 I do not have enough time for reading and assessment of evidence |  |  |  |
| 4.7 I do not have enough skills in presenting results of relevant research to my patients |  |  |  |
| 4.8 I do not have enough time to discuss research results with my patients during their scheduled appointment with me |  |  |  |
| 4.9 The use of EBM will further limit the number of patients that I can examine at my medical office |  |  |  |
| 4.10 Despite the results of relevant studies, patients have unrealistic expectations that influence  my choice of treatment |  |  |  |
| 4.11 I am concerned about the financial aspects of my practice because the access to EBM sources is costly |  |  |  |
| 4.12 Something else? *(write here)* | | | |

| **5. Understanding terms in EBM**  *(Choose one of possible four answers and cross "x" in appropriate box for each term)* | It would not be helpful to me to understand | Don't understand but would like to | Some understanding | Yes, understand and could explain to others |
| --- | --- | --- | --- | --- |
| STUDY DESIGN |  | | | |
| 5.1 Meta-analysis |  |  |  |  |
| 5.2 Randomized controlled clinical trial |  |  |  |  |
| 5.3 Cohort study |  |  |  |  |
| 5.4 Case control study |  |  |  |  |
| 5.5 Cross-sectional study |  |  |  |  |
| 5.6 Case report |  |  |  |  |

| **5. Understanding the technical terms EBM uses - continuation**  *(Choose one of possible four answers and cross "x" in appropriate box for each term)* | It would not be helpful to me to understand | Don't understand but would like to | Some understanding | Yes, understand and could explain to others |
| --- | --- | --- | --- | --- |
| STATISTICAL TERMS |  | | | |
| 5.7 Mode |  |  |  |  |
| 5.8 Median |  |  |  |  |
| 5.9 Interquartile range (IQR) |  |  |  |  |
| 5.10 Standard deviation (SD) |  |  |  |  |
| 5.11 Precision and accuracy |  |  |  |  |
| 5.12 Representative sample |  |  |  |  |
| 5.13 Test power |  |  |  |  |
| 5.14 P-value |  |  |  |  |
| 5.15 Confidence interval (CI) |  |  |  |  |
| 5.16 Type I and type II errors |  |  |  |  |
| EPIDEMIOLOGICAL TERMS |  | | | |
| 5.17 Odds ratio (OR) |  |  |  |  |
| 5.18 Relative risk (RR) |  |  |  |  |
| 5.19 Absolute risk (AR) |  |  |  |  |
| 5.20 Number needed to treat (NNT) |  |  |  |  |
| 5.21 Sensitivity and specificity of the test |  |  |  |  |
| 5.22 Heterogeneity |  |  |  |  |
| 5.23 Publication bias |  |  |  |  |
| 5.24 Positive predictive value |  |  |  |  |
| 5.25 Hierarchy of evidence |  |  |  |  |

| **6. Participant personal data** | |
| --- | --- |
| 6.1 Age *(write)* | ______ years |
| 6.2 Sex *(select)* | Male Female |
| 6.3 Work experience *(write)* | ______ years |
| 6.4 Have you specialised family medicine? *(select)* | YES NO |
